# Treatment resistant depression in the UK: sub-analysis of a European real-world evidence study

**DOI:** 10.1192/bjo.2021.192

**Published:** 2021-06-18

**Authors:** Jordan Talbot, Donald J MacIntyre, Shanaya Rathod, Joachim Morrens, Allan H Young

**Affiliations:** 1 Janssen UK; 2 Division of Psychiatry, University of Edinburgh, Kennedy Tower, Royal Edinburgh Hospital; 3 Southern Health NHS Foundation Trust, Research Department, Tom Rudd Unit; 4 Janssen EMEA; 5 Institute of Psychiatry, Psychology and Neuroscience, King's College London, Department of Psychological Medicine, London; South London and Maudsley NHS Foundation Trust, Bethlem Royal Hospital, Beckenham

## Abstract

**Aims:**

Treatment resistant depression (TRD) affects ≤20% of patients with major depressive disorder and is defined as failure to respond to ≥2 different antidepressants in the same major depressive episode (MDE). TRD patients’ outcomes are poor and real-world data from the UK are limited. The Treatment Resistant Depression in Europe Cohort was established to study patients being treated in local, routine clinical practice. The analysis presented here aimed to compare UK-specific data with data from other European countries included in the study.

**Method:**

A prospective, multicentre, observational cohort study of TRD patients in Italy, Germany, Spain, Portugal, the Netherlands, the UK and Belgium was conducted. Patients aged 18–74 years with current TRD, Montgomery-Åsberg Depression Rating Scale (MADRS) score ≥20, and initiating a new treatment for depression, were eligible. Data from medical records, clinician assessments and patient-reported questionnaires were collected over time, with follow-up of ≥6 months.

**Result:**

Data from 411 patients were analysed. At baseline, UK patients (n = 49) had similar depression severity to the whole European cohort (34.7% vs 32.6% of patients categorised as severe based on MADRS score, respectively). Patients had experienced the current MDE for a mean (standard deviation [SD]) of 6.1 (7.9) years vs 2.6 (3.9) years and 14.3% vs 4.9% had experienced ≥5 treatment failures during this time in the UK and whole cohort, respectively. Total mean (SD) Sheehan Disability Scale (SDS) scores of 24.5 (5.1) and 22.4 (5.5) were reported for the UK and whole cohort, respectively. Unemployment and long-term sick leave rates were 38.8% and 20.4% in the UK and 30.2% and 19.0% in the whole cohort, respectively. At 6 months, 8.9% of UK patients were in remission, and 82.2% had not responded to treatment, representing the lowest remission and highest non-response rates across all countries.

**Conclusion:**

UK patients had been ill for longer and had more prior treatment failures than other countries in the study. They had high work and functional impairment, and the worst treatment outcomes of all the countries studied. UK TRD patients experience high disease burden; there is an unmet need for treatment strategies with better response rates.

**Acknowledgements:**

We thank all participating patients. Study, and medical writing (Costello Medical, UK), funded by Janssen. AHY's independent research is funded by the National Institute for Health Research. The views expressed are those of the authors and not necessarily those of the NHS, the NIHR, or the Department of Health.

